# The Hospital. Nursing Section

**Published:** 1901-10-12

**Authors:** 


### The Hospital.

### Nursing Section.

Contributions for this Section of "The Hospital" should be addressed to the Editor, "The Hospital" Nursing Section, 28 & 29 Southampton Street, Strand, London, W.C.

No. 785.—Vol. XXXI. *SATURDAY, OCTOBER* 12, 1901.

### Notes on News from the Nursing World.

### THE FEATURE OF THE BUFFALO CONGRESS.

This week we give the concluding portion of the report by our special correspondent of the proceedings at the International Congress of Nurses, and it is now possible for our readers to judge how far British nurses were represented on the occasion. The Congress was in many respects successful. The address of the President, Miss McIsaac, was remarkable, alike for its generous tribute to English nurses and for its admirable lucidity. If the facts in Miss Stewart's paper on "Hospital Administration in Great Britain" were not new, they were excellently put together, and no attempt was made to exalt particular institutions at the expense of others. Miss Mollett pointed out some of the strong, as well as some of the weak, points of "County Hospital Administration." Miss McGahey dealt in a sufficiently exhaustive manner with the genesis and development of the Australian Association of Nurses. Miss Amy Hughes treated "The Origin, Growth and Present Status of District Nursing in England" at great length and with much ability. Though Miss Honnor Morten was not able to attend the Congress, a New York nurse read a hopeful contribution from her on the prospects of "The School Nurse." Miss Annie Arkle, a member of the Indian Army Nursing Service, described the conditions of the service, and added some opinions of her own, which, in view of the reorganisation of the Army Service, are timely at the present moment. There were also several animated discussions, and we are quite sure that our special correspondent correctly speaks of the social side of the Congress as "a feature." But a yet more striking feature was the absence of British nurses. America was, indeed, strongly represented by some of the most gifted nurses in that country, and, of course, they had not the same difficulties to overcome in the shape of distance and, leave a snurses from other parts of the world. Allowing for these, however, the paucity of representatives of the profession from this country must have impressed their American sisters. It is also brought out by the fact that Miss Stewart and Miss Mollett were the only matrons from Great Britain who addressed the meeting, Mrs. Strong, matron of the Glasgow Infirmary, being unfortunately prevented by illness; and still further by the circumstance that two papers were read on two successive days by Mrs. Bedford Fenwick. In short, Great Britain was almost as insufficiently represented at the Congress as South Africa at the preliminary meeting of the International Council of Nurses, when Miss Margaret Breay, honorary secretary and treasurer of the Matrons' Council of Great Britain and Ireland, essayed to speak in the name of the Dark Continent. For this, if for no other reason, British nurses are free to disclaim all responsibility for the resolution of the Congress in favour of State examination and public registration to which a handful of ladies from this side of the Atlantic endeavoured to commit them.

### AN ADDRESS AND A MEDAL.

Considerable comment has been excited in Maritzburg by the compliments paid to a young lady who went out to South Africa in the interests of a wounded relative, and subsequently was taken on as a nurse at the Assembly Hospital for three or four months. After she left the hospital she was presented, through the Agent-General for Natal, with an illuminated address by some of the patients, and on the 29th of July she received, with many others, the South African war medal from the King. Although, beyond a voyage home on a transport ship, she does not appear to have enjoyed any other nursing experience than that afforded at the Assembly Hospital shortly before it closed, she is described as a sister, with the result that some of the nurses who have worked hard and long in South Africa are saying that "honours are easily won, and sisters are easily made." The young lady is in no way to blame for the remarkable manner in which her services have been acknowledged, but great care should be taken at head quarters not to make honours too cheap.

### A RESERVE SISTER ON MILITARY NURSING.

The view of Nursing Sister L. M. Green on the military nursing, given in another column, are vigorous and somewhat original. It is amusing that, while she refuses to read anything that is written against the existing system, and sees no necessity for a nursing department, the reorganisation of the former and the creation of the latter has been decided upon by a strong committee. But it is always refreshing to meet with people who have the courage of their convictions, and Miss Green's recital of her experiences in South Africa will tend to confirm the opinion that things have not been so black as they were painted in some quarters.

### QUESTIONS FOR THE NEW NURSING BOARD.

In a criticism of the scheme for the reorganisation of the Royal Army Medical Corps, Mr. Burdett-Coutts, M.P., observes that the question of the employment of civil nurses "is left entirely to a future body to settle." He also notes in particular that "nowhere in the scheme is it stated or suggested what the strength of the nursing service is to be, nor is any instruction given to the future body as to what amount of female nursing a hospital is to have." At present, he points out, the manual says eight nurses to 520 patients, and adds, "Can it be claimed that the principle of female nursing is accepted when there is no specific review or reform of a regulation which for 50 years has been allowed to blot out the memory of Florence Nightingale from the records of the War Office?" But these questions can be dealt with by the Nursing Board, which will really be more competent to decide them than a committee composed exclusively of men.

### GRATUITIES TO DISABLED NURSES.

At a meeting of the Metropolitan Asylums Board on Saturday, the Hospitals Committee brought up for consideration, the question of a gratuity to a charge nurse who had been rendered unfit for further service, through contracting enteric fever whilst discharging her duties. It appears that Miss Henrietta Phœbe Bunyan, now aged 30, joined the Brook Hospital as charge nurse on May 1st, 1897. On May 15th, 1901, she was warded with an epileptoid seizure, and on June 12th, 1901, the medical superintendent reported that she would require a prolonged rest before she was fit to resume nursing work. He further stated that the nurse had a severe attack of enteric fever, complicated with phlebitis of the leg, in November 1898, and since then had not been as robust as before. He expressed doubt as to the advisability of retaining her services. The committee had, therefore, given her a month's notice, terminating her appointment. Miss Bunyan is entitled to receive a sum equal to the amount of her contributions under the Poor Law Officers' Superannuation Act, amounting to £5 18s. 4d.; but, in consideration of the circumstances of the case, the committee asked the managers to award her a gratuity for loss of office under Section 8 of the Act, and recommended that, subject to the approval of the Local Government Board, she be awarded a gratuity equal to the amount of one year's salary and emoluments—viz., £68 2s. 10d. The report, together with the recommendation, was adopted without discussion.

### POOR-LAW NURSES.

We are glad to see that "Another Poor-law Nurse" protests against the sweeping opinions of "A Poor-law Nurse" in our last issue. Both of our correspondents are practically acquainted with nursing in workhouse infirmaries and have a right to be heard. But the array of formidable and offensive adjectives applied to the poor-law nurse "as a rule" by "A Poor-law Nurse" seem to us to be an exaggeration of facts, and the irresistible conclusion is that she has been singularly unfortunate in her experiences. On the other hand, if we were at liberty to name the institution to which our correspondent who writes on the subject this week is attached, her views as to the system of nursing in the larger and well-ordered poor-law infirmaries would not occasion any surprise. She has the advantage of working in an infirmary which challenges comparison with the best-managed general hospitals, and under the head of one of the best of training schools. There is no doubt that the remedy she prescribes for the present unsatisfactory state of things—the disconnection of the infirmary with the workhouse—is admirable, but even then we fear that the poor-law nurse could not always be a paragon. "Fast, flighty, drunken, lazy" she need not in any circumstances be, and we hope and believe seldom is. Yet, even with a system as admirable as that under which "Another Poor-law Nurse" writes, there are sure to be more or less incapable, indifferent nurses, just as under the system she rightly condemns there are many most capable and devoted.

### "HANDY WOMEN" AT WORK IN IRELAND.

Some extraordinary statements were made at an inquiry held in the Kilkelly Dispensary by Sir Acheson M'Cullogh, Local Government Board inspector, into the necessity or otherwise of appointing a duly qualified midwife for the district. Dr. M'Cann, medical officer of the district, who contended that the appointment of a qualified nurse was an absolute necessity, said that "he knew several instances where mothers and infants were lost owing to the ignorance and incompetence of the country nurses, or, as they were styled, 'handy women.' Such nurses did not know it was necessary to call in a medical man." Dr. M'Dermott, medical officer for an adjoining district, confirmed Dr. M'Cann's statement and instanced several deaths of mothers and infants through the ignorance of the "quacks," who absolutely knew nothing about confinement cases. "Women of this class had infested the country, and when one of them knew a little of the business in her own peculiar way she was most dangerous, as she exercised her knowledge too far and very often with fatal results." Several members of the Swinford Board of Guardians also gave evidence showing the urgent need of the appointment, and at the close the inspector intimated that he would report to the Local Government Board in due course. Of course, the Board will sanction the required appointment, but it is also imperatively necessary that vigorous steps should be taken to prevent the "handy women" from "exercising" their knowledge—or want of it.

### SHIFTING THE RESPONSIBILITY.

The Walsall Board of Guardians have now formally decided to engage "an extra charge nurse for the infirmary." At the meeting at which this decision was recorded, they threw the responsibility for the insufficient nursing staff upon the medical officer and the superintendent nurse, whom they accused of not having asked for further help. But how can this be so when the medical officer in his report, to which we referred on September 14th, stated that the nursing staff was "quite inadequate?" We are really surprised that the Walsall Guardians have not also tried to shift upon the medical officer and the superintendent nurse the blame for the sanction given to the domestic servants of the infirmary to wear uniforms similar to that of the nurses.

### IMPROVEMENTS AT BELFAST.

The Belfast Board of Guardians continue to show signs of grace. Having passed a resolution to the effect that all probationers in their employ who during their first year of office contract infectious disease in the discharge of their duties, be granted a bonus of £5 upon their recovery, and if during their second year of service a bonus of £3, they decided at a subsequent meeting, by a majority of three, to erect a nurses' home in connection with the fever hospital. They have even determined to extend the weekly diet scale of the infirmary to the hospital nurses, and to give one chicken to each hospital nurse weekly, instead of one chicken between four nurses.

### NEW NURSES' HOME AT BOLTON.

The Bolton Board of Guardians are to be congratulated upon the new nurses' home which has just been opened under their auspices. It has a frontage of 184 feet, is in three storeys, and accommodation is provided for 40 nurses, with a separate bedroom for each. The whole are suitably furnished, and the nursing staff attached to the Union hospital will in future have no reason to complain that their quarters are not comfortable. At the opening ceremony a brass tablet commemorative of the event was unveiled, and among the company was Mr. H. Jenner Fust, of the Local Government Board, who dwelt on the value of separate nurses' homes in connection with Union hospitals.

### NURSING IN AN AMERICAN ISOLATION HOSPITAL.

In a paper on an American isolation hospital read before the Massachusetts Association of Boards of Health, Dr. May Holmes, superintendent and resident physician of the isolation hospital at Worcester, states that the question of obtaining nurses has throughout been a serious one. At first the authorities depended upon the City Hospital, and a course in contagious diseases was made a part of the curriculum of their school. But as time went on it became impossible for them to furnish a sufficient number, and they were obliged to find nurses from other sources. These sources, however, dried up, or were not satisfactory, and the authorities were driven, as a last expedient, to train their own nurses. They propose to supplement the course in the isolation hospital by work elsewhere in surgery and obstetrics, and this will be done before any certificate is awarded. Dr. Holmes mentions that the fear of contracting the diseases has had its influence more or less upon the peace of mind of the nurses. She continues: "A certain proportion, perhaps half, have been immunized with diphtheria antitoxin. No one of these contracted the disease under six weeks' time. The rashes have been rather disagreeable in some cases, and we have, therefore, ceased to urge the immunization. Should a nurse contract the disease we have no fear of being unable to control it with the help of anti toxin." It is interesting to learn that since the hospital was opened 97 nurses have been employed, and only nine of them have contracted disease—namely, four diphtheria, four scarlet fever, and one whooping cough.

### COTTAGE NURSES IN NORFOLK.

Lord Lindley made a speech the other day at a meeting held in Hillington Hall under the auspices of the Norfolk District and Cottage Nursing Federation, which calls for comment. Admitting that it was necessary to grasp what was meant by cottage nursing, he proceeded:

> The cottage nurse must be a cottager—one who would not turn up her nose at living with cottagers and doing cottagers' work. Next, she must be trained; and to train a young woman to be a cottage nurse was by no means easy. The difficulty lay in the fact that as she was trained her expectations rose, and her standard of living was raised; and there was a danger that she would become too big for her shoes. If she were trained to be the equivalent of a district nurse or a higher-class nurse, then there would be a difficulty in getting her to do cottage work. This was a practical difficulty, and one of the greatest to be overcome—to get a trained nurse willing to lead the life of a cottager.

Lord Lindley went on to say that, in spite of this difficulty, some suitable individuals had been found. But the six months' "training" which he urged was essential, adding that if "the nurse had been to service and knew a little of housemaid's work, so much the better," does not convert the cottage nurse into a trained nurse, however willing she may be to lead the life of a cottager. There is no objection to the employment of a cottage nurse if the services of a district nurse—who, as Lord Lindley said, is "a totally different kind of person"—cannot be obtained; but she must not be permitted to masquerade as "a trained nurse" on the strength of a smattering of nursing knowledge.

### NURSING IN RHODESIA.

It is announced that the Dominican Sisters at Salisbury Convent, who constitute the nursing staff of the local hospital, are resigning their duties as nurses. Whatever may be thought as to the policy of placing the nursing in an ordinary hospital in the hands of a religious order, there is no doubt that the devotion and self-sacrifice of the Dominican Sisters, who have been in Salisbury since the occupation of the country, have created a most favourable impression, and that they are justly respected. In future, we understand, they intend to devote themselves to education by the development of boarding schools for girls and boys in Rhodesia.

### SISTERS AND ORDERLIES.

Army sisters at the front will be interested to note the activity which the War Office is showing just now in recruiting for the orderly section of the R.A.M.C. From the reports of some of the nurses who have returned from South Africa it is evident that many difficulties have arisen in dealing with the raw material which takes the place in the A.N.S. of our hospital probationers. The latest recruits are to be enlisted under the old arrangement, for a period of one year, or for as long as the war may last. They must possess a fair knowledge of ambulance and nursing duties, and, according to the Army Order, they are to be paid 3s. a day.

### RESIGNATION OF A MATRON.

Miss Kathleen Disney, Lady Superintendent of the Victoria Hospital for Women and Children, at Cork, having been ill for several weeks, has, we understand, thought it well to resign her post, and go home for a long rest.

### SHORT ITEMS.

The *Orient* arrived at Southampton from South Africa last week, with Nursing Sisters E. K. Hamilton, L. M. Green, M. Pedler (all returning when leave expires), A. B. Joel and M. Sabey (both resigning), on board. The *Nubia*, arriving at Southampton on Tuesday, had on board Nursing Sisters D. F. Palmer and J. E. Phillips (returning to ship); E. M. Beetham and E. T. Wells (returning to South Africa after a month's leave); A. J. Douglas, M. E. Hainnelin, and E. C. Rider (Imperial Yeomanry Hospital, time expired); M. L. Gordon and G. Nutter (resigning).—A series of sixteen lectures, which are free to all nurses, will be delivered fortnightly at the City Orthopædic Hospital, Hatton Garden, on Fridays at 4.30 p.m. Mr. Noble Smith gives the first two, the second being on Friday, October 25th, on "The Management of Curvatures of the Spine."—The Hammersmith Progressive Benefit Society is getting up an entertainment in aid of the funds of the District Nursing Association of Hammersmith and Fulham, to be held on the 24th inst.

### Lectures to Nurses on Medicine and Medical Nursing.

By Norman Dalton, M.D. London, F.R.C.P., Physician to King's College Hospital.

### LECTURE XXI.—DISEASES OF THE NERVOUS SYSTEM.

The nervous system is divisible into the "cerebro-spinal system," which consists of the brain, the spinal cord, and the peripheral nerves; and the sympathetic system, which consists of a number of ganglia (collections of nerve cells) and nerves. Diseases of the nervous system, like diseases of most organs, are divided into two classes: (1) organic diseases, and (2) functional diseases. In organic diseases there is some alteration in the structure of an organ which can be detected with the naked eye or with the microscope. For instance, a tumour in the brain is an organic disease. Another instance is a condition called "sclerosis," in which the tissue of the brain or cord becomes hard, grey, and shrunken. The actual change in the tissues in an organic disease is often called the "lesion." On the other hand, a functional disease is one in which we are unable, even after the most minute examination, to detect any change in the tissues. Functional diseases of the nervous system are called néuroses. A good instance of a neurosis is epilepsy, for although convulsions occur during life nothing to account for them is found after death. Many functional diseases are caused by some poison which circulates in the blood, but it is probable that others are associated with abnormal action on the part of the sympathetic nervous system. The sympathetic nerves supply the walls of the blood vessels and also, to a certain extent, the heart. Consequently they regulate the blood supply to the organs. If they constrict the blood vessels the organ gets less blood, and if the blood vessels dilate it gets more blood, and such changes in the amount of blood it receives is known to alter the functions of many organs. The same nerves supply the muscular walls of the whole alimentary canal, the muscular coat of the bronchi, the uterus, etc. The sympathetic system is greatly acted upon by the emotions; for instance, fear may cause an irregularity of the heart's action; or may excite diarrhœa, because the sympathetics set up contractions of the muscular coat of the bowels. The sympathetic system is not directly under the control of the higher nervous centres which lie in the brain, and in which are situated our mental capacity, our will power, etc.; but nevertheless the will power of a healthy person will enable him to control the actions which would naturally follow when his sympathetic system is excited. For instance, a soldier in action may be afraid, and he is quite unable to prevent his sympathetic nerves from producing pallor by contracting the arteries which supply the skin of the face with blood. His natural tendency would be to run away, but to do so he must use the voluntary muscles of his legs. Now the voluntary muscles are under the control of the higher brain centres, and if the soldier's will be strong enough he does not use these muscles for the purpose of running away, but keeps them at rest or uses them to advance according to the orders of his officer. It is obvious that, if a person cannot control the impulses which are excited by the emotions, it is either because his emotions are too powerful or because his higher centres are too weak. There are many persons who are born with a nervous system which soon becomes unstable, so that their cerebro-spinal system cannot control their sympathetic, and they are called "neurotics." The same instability may be acquired by intemperance, dissipation, etc.; but it is quite possible that the giving way to such indulgences is an indication that the nervous system of these people was unstable from the beginning of their life. Now there are other things besides the emotions which are apt to unduly influence the brain of neurotic persons. All healthy people are from time to time liable to uncomfortable sensations for which they are unable to account. It may be a pain in some part of the body, or a sensation as if the heart stopped for a minute, or a feeling of giddiness, etc. If the sensation be transient a stable-minded man rapidly forgets it. If it persists, he consults a doctor. If he is told that the sensation is not a sign of serious disease, he soon learns to treat it as a negligible quantity. He finds out from his doctor the best means of preventing or of removing the disagreeable sensation, and it soon ceases to have any influence on his life. Now it is quite different with a neurotic person. The unaccountable pain produces a deep impression on his mind, which dwells on it, magnifies it, and finally is able to bring it on by merely thinking of it. It is the same with the heart. The mind watches its action and detects the slightest irregularity, or imagines an irregularity which does not exist. Various other symptoms occur in these patients, the importance of which their minds exaggerate, and in bad cases the mind becomes so absorbed by the anxiety and alarm occasioned by symptoms which are really unimportant that the victims are unable to work or to partake in any amusements. Sometimes they become extremely debilitated, in which case the term "neurasthenia," or nervous exhaustion, is applied to their condition. Some of the persons who are debilitated from this cause remain plump, but others become emaciated, especially when the morbid sensations are present in the stomach or intestines. It must not be supposed that neurotic persons are feeble minded. It is that their minds are unstable, and their mental balance likely to be upset by some causes, but not by all. For instance, a man may possess courage and have gained the V.C., but a trivial irregularity of the heart may render his life useless. Neurotics, when the evil spirit is upon them, are a great nuisance to their relatives; but at other times their brilliancy of conversation, their musical talent, etc., may make them most delightful companions. There are some neurotics, however, who appear to have no redeeming qualities, and who at their best are incapable of doing any good work or of making themselves agreeable in any way. The worst cases are, of course, very difficult to distinguish from cases of insanity.

The object of what has been said is to make a nurse understand the nature of cases of hysteria and hypochondriasis. These affections are "neuroses," but (except for the fact that we know that they are not going to be fatal) the sufferers from them are as much objects of commiseration as if their troubles were organic. It is true that in some cases the mental condition is so low that the patients become untruthful, and say that they feel what they do not feel; but in the vast majority of cases the sensations are as real to the patient as if an organic cause for them existed. For instance, a woman with an hysterical knee feels the pain as acutely as if there were really disease of the knee-joint; and a man with depressing sensations in the epigastrium is as uncomfortable as if there was a cancer in the stomach to account for it. In many cases some real illness which has led the patient to overestimate the importance of passing sensations, or which has weakened the power of the higher brain centres, is the starting-point of the trouble; and in women hysteria can often be traced to the regular recurrence of severe menstrual pain, or the prolonged fatigue and anxiety attendant on the nursing of a relative. Such patients must never be told that there is nothing the matter with them. The nurse must encourage them and endeavour to distract their attention from their ailments. She must also be firm and insist on the doctor's regulations being carried out, while such sympathy as she gives must be of a cheerful and robust order. A doctor sometimes adopts a brusque manner, and I have seen a patient who said that she could not walk get out of bed promptly when sternly ordered to do so by a new doctor. But this plan is not always successful, and nurses must never attempt it. Bad cases must be taken away from their relatives and put under the charge of a nurse. They must be fed abundantly and be treated by massage and electricity. This method of procedure constitutes the "Weir-Mitchell" treatment.

### Nursing in South Africa.

By a Correspondent.

### A CHAT WITH A RESERVE SISTER.

Nursing Sister L. M. Green, on whom I called a day or two ago for a chat, began her conversation in an unexpected manner.

"My opinion of the R.A.M.C. has gone up many degrees," she said, "the devotion of the orderlies with whom I have had to do has been something astonishing. Day after day they have done their utmost, over and above their regulation duties, for the patients in hospital."

"You had no trouble with drunkenness?"

"Personally, none whatever. And the stories one hears about such troubles usually come from the same source; I mean, if once a sister has a disorderly set of men, she is likely often to have them. The fact of the matter is, that so much depends on the personality of the sister. If you are good to your orderlies they will be good to you. A little interest in their meals, or a cup of hot cocoa in the middle of the night, will make a great difference to the way the work is done. They have to put up with a great deal of 'roughing it,' and they get very little praise. Of course you must be very particular, and see that things are properly done, and you must never let anything pass."

"You have nothing to say against the system?"

"No. Mr. Atkins is very well looked after. I have very little patience with people who complain of military nursing; in fact, none at all; and I absolutely refuse to read anything that is written against it. People in England have no idea of the way these criticisms preyed upon the sisters' minds in places like Bloemfontein. My opinion is that it's a waste of time and energy to discuss the matter; there the system is, and we have to make the best of it. I consider it a very good one."

"You do not see the necessity for a new nursing department?"

"No, because it is there already. When I was at one of the home stations, there were ten-minute lectures to the men every morning; and these were given not only by the doctors, but by the sisters also. Each sister had to train her own orderlies; and the subject was set each day—how to wash a patient, how to make a bed, and so forth. Of course it was impossible to train them in South Africa, but then it was not necessary, because they had received their training at home, quite as truly as a probationer in a civilian ward, even in a good hospital; for more is picked up than actually taught."

"Public criticism has shown that the nursing staff was not large enough," I remarked.

"Of course there were not enough army sisters, but there was the reserve," and Sister Green went on to tell me how she herself became a member of the A N.S.R.

### Joining the Reserve.

"I was trained at Barts," she said, "and when I had finished I took an appointment under the Order of St. John of Jerusalem. Sir John Furley then asked me if I would join the reserve, in case war broke out and my services were required. At first I refused, saying, that if I had wanted army work I would have gone in for the regular army training. However, I thought better of it; there was no prospect then of the war in South Africa, and I sent to St. Bartholomew's for my certificates. Sir John Furley told me not to be in a hurry, and it took me six months, I think, to read through all the regulations and get everything settled. If I had joined at once I should have been the 99th; as it was, three others joined before me, and I was the 102nd. War broke out very soon afterwards; I was called up in my turn and I have been doing military work ever since."

"Do you like the work?"

"Yes, I love it, and I was proud to go and do my part. I went out with No. 10, leaving England on the 12th of March 1900, on board the *Avoca*. We arrived on April 10th, and I was with No. 10 at Gray's College, Bloemfontein, until the 1st of June, when I was asked to take charge of the sisters who were ill at Bishop's Lodge—the house belonging to the Bishop of Bloemfontein. It was a pretty house surrounded by greenery, and with a verandah on which were several convalescents, while two or three stood on the steps.

"How did you like nursing the sisters?" I asked.

"It was very hard work, because no one had time to help much. There were sixteen ill, and when I say that they had to be washed and got ready for the doctor by nine o'clock, you will see that there was plenty to do. I refused to go out at all until help arrived; it was very responsible work, but I am glad to say they all recovered. There has been one death since I was there, I hear."

"Were they mostly cases of enteric?"

"Yes, and a few had dysentery. In November I went back to Gray's College to take charge of the officers' ward on the upper floor."

### Enteric Notes.

"It is most extraordinary," continued Sister Green, "how the enteric acts on some of the men. The most curious thing is that they get it over again. We had one case in which there was first a relapse, then a period of convalescence for, I should think, quite six weeks, and then the patient had a second attack. The doctor said he couldn't call it a relapse so long afterwards; and all the original symptoms were gone through a second time. Another remarkable thing was the number of cases of thrombosis after enteric. They must be, I think, the result of the long marches the men have gone through, and I can't help feeling that perhaps it is partly due to the quantity of stimulants it is necessary to give enteric patients in that climate. For such cases rest was the great thing of course."

"Did you remain long at Gray's College?"

### Nursing Under Canvas.

"I left on June 8th. There was a chance of getting up the country, and as seniors have the first chance, I was asked if I would like to go to Johannesberg. I did so, joining No. Thirteen General Camp, under canvas. I can honestly say that it is an ideal hospital. I have never seen anything better managed; the theatre is splendid, quite up to date, with glass-topped operating table and standard irrigators, etc. It was at No. Thirteen that my opinion of the R.A.M.C. went up so much. There were many days when the staff did more than merely go their rounds according to rule; and often I have known them stay behind when really off duty to see if there was anything extra to do."

"How was the nursing arranged?"

"There were six patients in a tent, and five E. P. tents in a line; four lines in all, with a sister to each. The worst cases, requiring most frequent attention, were put at one end of the line; this saved the sisters' time, and was convenient in every way. Then as they recovered they were moved down the line. All the patients did well."

"Had you any surgical cases?"

"Yes, after the last battle; some were ghastly sights, too. It was really like murder when men came in with wounds from a wrecked train or a chance shot from a sniping party. I prefer medical cases myself, especially enteric."

"Was there much illness on board the *Orient*?"

"Not a great deal. About 500 were invalided home, but only a small proportion required nursing; most were convalescent. There were several cases of malaria, and the rest were principally enterics."

"Are you returning to South Africa?"

"Yes, when my leave is ended. I love the veldt and the free life. When you are off duty you can do so many things. Riding is a great delight and quite possible in the afternoons. Perhaps I am rather Bohemian, but I have certainly quite fallen in love with a sister's life in South Africa."

### The Congress of Nurses at Buffalo.

By Our Own Correspondent.

(*Continued from page* 12.)

The following is the paper read by Miss S. B. McGahey, on "The Nurses' Federation of Australia," at the International Congress of Nurses at Buffalo with the report of the discussions at Thursday's sessions:—

Several months previous to the granting of federation to the Australian colonies, an Association of Nurses was founded in New South Wales, named after the colony in which it was inaugurated. The objects of the Association are:—1. To promote the interests of the trained nurse, male and female, in all matters affecting their work as a class. 2. To establish a system of registration for trained nurses. 3. To afford opportunities for discussing subjects bearing on the work of nursing. 4. To initiate and control schemes that will afford to nurses a means of providing an allowance during incapacity for work caused by sickness, accident, age, or other necessitous circumstances. A few months after its inception it was considered advisable to change the name to a more comprehensive one in view of the fact that so many nurses trained in the other colonies had been enrolled as members. This was accordingly done at a special meeting convened to deal with this and other important business, and the alteration met with general approval. Since that date it has been known as the Australasian Trained Nurses' Association. The Association was not established until 1899, but the nurses were active during the years preceding. The Australasian Trained Nurses' Association is managed by a council consisting of a president, a vice-president, honorary treasurer, two honorary secretaries, and seventeen members, of whom five are duly qualified medical practitioners, five matrons and superintendents of nurses, five sisters and nurses, and two honorary members. Since April 1st, 1900, the following conditions have had to be complied with by applicants for membership: (*a*) That they have been engaged for three years in general hospitals recognised by the Council and containing not less than 40 beds; or (*b*) That they have been engaged for four years in a country, district, or suburban hospital recognised by the Council and containing not less than 20 beds, and have been trained at that hospital under a matron or nurse who holds a certificate from a training school for nurses recognised by the Council of this Association; or (*c*) That they have been engaged for five years in a private, country, district, or suburban hospital recognised by the Council and containing not less than 10 beds, and have been trained at that hospital under a nurse or matron who holds a certificate from a training school for nurses recognised by the Council of this Association. Furthermore all candidates must give reference as to their moral character, produce certificates of competency from the hospitals in which they have been engaged, and account for any interval of time in their nursing career when not engaged in hospitals or nursing associations. At present 406 registered nurses are enrolled; besides these 64 medical practitioners have joined the Association, amongst whom are the leading physicians and surgeons of Sydney. Of the registered nurses outside New South Wales, 17 are in Victoria, 18 in Queensland, four in Tasmania, three in South Australia. No doubt branches in several of the States will soon be formed, as the nurse members in many of those States are anxious to have local centres established. An auxiliary branch of the midwifery nurses in connection with the association was formed during the latter part of 1900, and up to the present date 50 members have been enrolled. These midwifery nurses have a separate register, and are entitled to attend at the general meeting of the association to elect one of their members to represent them on the council of the association. In this country a large majority of midwifery nurses have not had any general training before taking up this speciality. During the past few years many more general nurses have studied this branch of nursing than was customary previously. The qualification for membership is a certificate proving that the candidate has received six months' instruction in practical and systematic training at one of the maternity hospitals in Australia recognised by the council of the A. T. N. A. The candidate is also required to have passed a satisfactory examination before a competent board of examiners. Candidates trained in European or American maternity hospitals in which the course of training is equivalent to the standard required in the Australian hospitals are eligible for membership. In July, 1900, a bevevolent fund was established, and although the sum subscribed is small, hopes are entertained that it will be increased. Three of the members of the association who have private hospitals have each put a bed at the disposal of the council for any nurse member who may require medical or surgical treatment. Soon after the inauguration of the A. T. N. Association a society of nurses was formed in connection with the Prince Alfred Hospital Training School, Sydney. This society was named the Prince Alfred Hospital Trained Nurses' Reunion, the greater number of whose members is also on the register of the association. The list of members has now reached 100. As each class graduates new members are enrolled. The conditions under which the nurses work have much improved. Modern hospitals have been erected, and nurses' quarters, replete with all the necessary comforts, are now established in connection with most of the leading hospitals. The daily average off-duty time for pupils and nurses is three hours; in some hospitals two days' holiday in the month are given besides. The annual holiday is two or three weeks. An eight hours' day for nurses has not yet been introduced in any of the hospitals in the Australian States, but in a few of the New Zealand ones this system has been in force some time. The course of training in the larger schools is for three years. At the Prince Alfred Hospital and Sydney Hospital the period has been extended to four years. The curricula varies in the different schools; in some the course of instruction has been considerably increased. At Prince Alfred Hospital, midwifery nursing, dispensing, and housekeeping have been added, and it is proposed to deal with those subjects during the fourth year of training. The non-payment system does not exist to any extent in the Australian training schools; in a few a premium is required to be paid by the pupils on entrance, but the majority give some remuneration to the pupils during their period of training.

### Preparatory Instruction for Nurses.

Thursday's sessions were shortened in respect to the final funeral and burial services of President McKinley at his old home in Canton, Ohio.

Mrs. Rebecca Strong, superintendent of nursing, Glasgow Infirmary, was down on the programme for a paper on the subject, "Preparatory Instruction for Nurses"; but the Secretary announced that Mrs. Strong was ill in Philadelphia and unable to attend. The Congress sent a telegram of regret to Mrs. Strong, and a discussion of the subject was taken up by the delegates.

Miss L. L. Dock, secretary of the American Society of Superintendents of Training Schools, followed with a paper on "The Three Years' Course." The success of the three-year course, she said, is absolute. Through it the hospital service is greatly improved, and instead of lessening the number of desirable applicants, the three-year course increases the number. The three years also allow better grading of the course, and advances toward a more highly developed course is made. More attention is given to the housekeeping and dietetic basis of nursing, and the hours of work can be more readily arranged. The question of private nursing by undergraduates was introduced by Miss Dock, and formed the basis of the discussion following her address.

Mrs. Bedford Fenwick said that, 25 years ago, when she took a training course, it was of only one year's duration. Later, it was extended to two years, then to three, and in some cases in England the course is now four years. She approved of the larger courses.

Miss Stewart, matron of St. Bartholomew's Hospital, said that in her hospital the regular course is three years, with the provision that the certificated nurses should remain at the school an additional year. As far as she had observed, she said, the American way is, when they have done a thing, to set at once about it to improve it. The English way is, when they do a thing, to sit down and look at it. "I am not sure," she concluded, "that the English way is not the best. That fourth year in the hospital after graduation is really the 'looking at it'—the contemplation of their work. It deepens the life and character of the nurse. It makes a woman of her. It gives her time to arrange her experience, and put it a little to the test. The nurse gets a small salary in the fourth year, and when she so agrees, goes out private nursing during that time."

Miss C. J. Wood, managing director of the Nurses' Hostel, spoke in favour of the preparatory instruction for nurses. Crowding the first year's practice and theory together when the probationer is fresh in the work is often the cause of the breaking down of the nurse.

Miss Mary A. Snively, of Toronto, and Mrs. Hunter Robb, of Cleveland, endorsed Miss Wood's opinion.

A paper on "The Nursing of the Insane," by Miss Laird, Superintendent of Nursing in the New York Hospital for the Insane in Seneca County, was amplified by Miss Richards, of the New England Hospital for Women and Children, and by Miss C. J. Wood.

### Private and Hourly Nursing.

Mrs. Rogers, Superintendent of Bridgeport Hospital, Conn., and Miss Carr, of Baltimore, treated the subjects, "Private Nursing" and "Hourly Nursing" in their addresses. Hourly nursing is a comparatively new branch of the profession in America, and there was a great deal of interest shown in the discussion.

Miss Carr said: The need for visiting or hourly nursing has, I am sure, been brought home to us all by the instances coming under our personal observation. Self-supporting women in boarding-houses, houses where trained care and advice is sorely needed, but where the family resources do not permit of an outlay of twenty or twenty-five dollars a week. In London, a long time ago, talking to an old lady of nearly eighty who knew Florence Nightingale, who during her whole life had been associated with many and varied forms of philanthropy, I mentioned the plan of hourly nursing which had been begun and she told me that in her opinion it was one of the most hopeful signs in a profession at whose development she was inclined to shake her head. But the chief argument in favour of this kind of nursing is that it brings the services of the trained nurse within the range of nearly all wage-earners, and is a paid arrangement for supplying non-charitable help, the basis of the system being adequate remuneration for services rendered.

In this connection Fraulein von Vollenhoun, of Holland, one of the visiting nurses, gave her ideas on private and hourly nursing. She spoke in quaint, broken English, apologising for her imperfect knowledge of the language. All nursing, she thinks, will in time be done in the homes and not in hospitals. It keeps the home intact and the family together. If the mother is ill she is better to be attended at home, where she knows her children and husband are being properly cared for and is not anxious and distraught concerning them as would be the case if she were removed. If the home is poor or untidy, the coming of the nurse is an example to them. The family see her neat and tidy and benefit thereby. At this point Fraulein grew hopelessly lost in attempting to express her thoughts in English, and stopped. "I want to say much, many more," she said quaintly, "but you see how I can not." And the Congress applauded and called her again.

Miss Carrie M. Earley, a coloured delegate, matron of the Frederick Douglas Hospital in Philadelphia, spoke in favour of the three-year course. Another coloured delegate at the Congress is Miss Ida M. Lightfoot of the Coloured Home Hospital in New York. Both are skilled in their profession, and were well received by the Congress.

The afternoon session closed with a symposium on "Opportunities and Responsibilities of the Graduate Nurse of To-day," conducted by Miss K. De Witte of Chicago, Miss Richards of Taunton, Mass., and Miss Patton of San Francisco.

### District Nursing in England.

Friday morning's programme began with a paper by Miss Amy Hughes, late Superintendent of the Nurses' Co-operation, on "Historical Outline of the Origin and Growth and the present Status of District Nursing in England." This was followed by papers on "Methods and Progress of District Nursing in the United States," by Miss Harriet Fulmer, Superintendent of the Visiting Nurses' Association in Chicago, and "Nurses' Settlements," by Miss Wald, head worker of the Nurses' Settlement in New York. These papers described conditions of poor nursing in the two cities which are similar. This class of nursing in America is voluntary, the nurses making contributions of their services, or being paid by small organisations of charitable women and men.

An organised system was described directly afterwards by Miss McLeod, Chief Superintendent of the Victorian Order in Canada. So impressed were the American delegates by the system in Canada that in the discussion which followed the three addresses Dr. Flora Hughes, of Boston, suggested that the United States delegates should combine to form an association similar to the Victorian Order as a memorial to William McKinley, the late President. The suggestion was greeted with cheers.

### The Victorian Order in Canada.

Describing the Victorian Order, Miss McLeod said:—The pioneers in district nursing in Canada were the Sisters of Charity, who also led the way in hospital nursing. The Sisters of Providence have long been engaged in this work, especially in the Province of Quebec. Early in the year 1897, the year of the celebration of our late beloved Queen's Diamond Jubilee, the women of Western Canada recommended that an organisation be formed as a Jubilee offering to Her Majesty which would provide nursing service in the more remote districts of the Dominion. The Countess of Aberdeen responded enthusiastically, and when the project known as the Victorian Order of Nurses for Canada was finally launched, owing to the false rumours as to the aims of the Order, it met with little response from either doctors or nurses. This prejudice was eventually overcome and a Royal charter was procured, which authorised the formation of a board of governors which should have the management and control of the Order, and appointed His Excellency the Governor-General of Canada as patron. The Order undertakes to teach district nursing. Only nurses holding diplomas from some recognised hospital or training school, and who come highly recommended, are considered eligible for the course of training in district nursing. A period of four months' probation is given at the training home either at Montreal or Toronto to test the adaptability, tact, and previous training of these nurses. At the expiration of that time the nurse is recommended to the board of governors as a candidate for the Order. When she enters the Order she is pledged for two years' service, and must be prepared to go anywhere in the Dominion for district nursing or to serve in one of the cottage hospitals. She is provided with on outfit and receives a salary of not less than $300, with maintenance and laundry. The work of the Order has slowly but steadily progressed, and has been extended from coast to coast. Twenty-six branches have been established, and in all fifty nurses are engaged in the work. Her Excellency the Countess of Minto is doing much to create a special fund to provide suitable buildings for cottage hospitals. We have now seven cottages in the West, and these will be open to give a training in nursing to the educated Indian girl in order that she may be of special service to her own people. In the remote districts of the North-West the nurses often have to drive long distances or canoe up a river to reach the patient, and may have to remain a week. Thus the district nurses accommodate themselves to the conditions in both city and country.

### The School Nurse.

Miss Honnor Morten was unable to be present, and her paper was read by Miss Wald, of New York, as follows:—There is no daily medical inspection of primary schools in England, though the spread of both major and minor infections among the school children is fully recognised. While the State and municipal authorities are deciding what they shall do with regard to arresting this spread of disease, private individuals have been sending nurses to some of the schools in London, Liverpool, and elsewhere, and doing the work quietly as a charity. It was on this line that the London School Nurses' Society was founded, in 1898, with the Vice-Chairman of the London School Board as its chairman of committee. The staff of nurses has always been small—never more than five—though the elementary schools of London number over 500, and the nurses have only been able to visit the very poorest schools and touch the mere fringe of the evil. But they are getting a slow recognition by the authorities, a rapid regard from scholars, teachers, and parents. On February 27th, 1900, the following notice appeared in the School Board of London *Gazette*:—"The School Management Committee give their consent to a nurse from the London School Nurses' Society attending each morning for one hour and a half to dress the eyes and sores of the children in those schools where the divisional members consider it desirable, and make the necessary arrangements, provided that the Board shall not be liable for any of the costs thereof, and in any case where a school is visited by a nurse of the Society the Board provide a basin and kettle for the use of a nurse at a cost of 3s. for the two articles." The Government Inspector, in his report of April 19th, 1900, in Saxon Street School, said: "The visits of a nurse to this very large infants' school have proved most beneficial to the health of the children, so much so that it could be wished that the School Board might make such visits universal in their schools in poor localities." Then in June of this year, 1901, the School Board cautiously appointed one nurse of its own as an experiment, especially to deal with a virulent form of ringworm which is very prevalent. This nurse is not yet at work, but the fact remains that for the first time the public school authority in England has made such an appointment. The work done by the voluntary nurses consists in weekly or, if necessary, daily visitation of the schools, seeing the children sent to her by the teachers, dressing small sores, cleaning dirty heads, and bathing sore eyes. Where necessary the teacher is advised to exclude a child, or a bad case is followed to its home, or the mother sent for to be seen at the school. It is found that pediculi and mild ophthalmia form the large majority of the cases. In some girls' schools as many as 90 per cent. of the scholars were found to be suffering from pediculi. After six months' regular visiting these head and eye chronics become cured, and a higher standard of cleanliness reigns in the school, and the nurse's visits are less frequent, and often very brief. The ideal would be for some six school nurses in a district to visit daily under a doctor, reporting to him where major infections, such as diphtheria, were found and medical help wanted, while they attended to the enormous number of cases of minor infections. Towards such an ideal we are slowly but surely tending, and then the School Nurses' Society will gladly dissolve and hand over its charitable voluntary work into the hands of the school authorities.

At the afternoon session the first topic, "Organisation and Registration of Nurses," was treated in its general aspect by Mrs. Bedford Fenwick, of London, and specifically by Miss Sylvern Nye, speaking for New York State, and Miss Mary Agnes Snively, for Canada.

The last division of the afternoon was occupied by Mrs. Dita H. Kinney and Miss Annie Arkle on the general subject of army nursing.

### Army Nursing in the United States.

Mrs. Dita H. Kinney is Superintendent of the Army Nurse Corps of the United States Army, and the subject of her paper was "Army Nursing in America." It opened with a tribute to Florence Nightingale and General George M. Sternberg, Surgeon of the United States Army, and continued with an outline history of army nursing in the United States from 1775 to the present time, dwelling especially on the experience of the Spanish-American War nursing, the result of which was the enactment of the Reorganisation Army Bill on February 2nd, 1901, by the terms of which the Nurse Corps became a part of the medical department of the United States Army. The nurses are since appointed by the Surgeon-General with the approval of the Secretary of War. After appointment nurses are required to serve four months in the United States before being assigned to foreign duty. The term of service is three years, the term of foreign duty from one to two years. The medical department, she said, has given up the use of the Red Cross, and in its place adopted its own colour, green; but the Red Cross is still used on hospital flags, ships, and ambulances.

### The Indian Army Nursing Service.

Miss Annie Arkle, delegate from the Indian Army Nursing Service, read the following paper:—The Indian Army Nursing Service was initiated by that good friend of the British soldier, Lord Roberts, in 1888; and although it has been in existence barely fourteen years, there has been great advance in the understanding of nurses and nursing in India, and the necessity for nursing and caring for sick soldiers has been acknowledged. Candidates for the service must have had at least three years' training in a civil general hospital. The service is composed of lady superintendents, of whom there are four, and nursing sisters, of whom there are between fifty and sixty. The duration of the term of service is five years, after which time the sister is entitled to one year's furlough out of India on two-thirds pay, with home passage from and to her station. At the end of the five years she can leave the service or sign an agreement to return for another term. In the event of her leaving she will receive a gratuity of 500 rupees (about $150) after the first term, 1,500 rupees (about $450) after the second term. The gratuity given to a lady superintendent is proportionately higher. If she agrees to return she receives two-thirds pay while on furlough. Should she leave before her first term of service is completed, for any cause save sickness, she will be obliged to pay the sum of £25 ($125), or give six months' notice and pay £20 ($100). After 15 years' service the sister receives a pension of about $250 per annum. After 20 years' service this pension is increased to about $300, with an addition for every year's service as lady superintendent. In addition to free quarters, fuel, light, and punkah-pullers, the lady superintendent receives $300 a month; the nursing sister $175. When she becomes senior sister in a station (where there is no lady superintendent) she receives $200. There is always a small compensation allowance, varying with the rate of exchange. The lady superintendent has control over all the sisters in her command. Once every year she visits the nursing staff of all hospitals in her command for the purpose of inspection, and afterwards submits a full report on the manner in which each sister has done her duty, which reaches the principal medical officer of His Majesty's Forces in India through the prescribed channel. Should the report not be favourable it must be shown to the sister concerned, who has the opportunity of making an appeal, and has the right to have the matter inquired into by a board of officers in the usual way. The senior nursing sister does the housekeeping and is responsible that order and regularity be carried out in the quarters and in the wards. When a new sister arrives at a station she usually pays an entrance fee for the use of things we need in the quarters not provided by Government. This is very hard on a sister who is moved often from station to station, and much expense would be spared the sister if the Government would grant a small amount fully to cover these expenses. The hours of the sisters on duty vary in some stations. As a rule there are three sisters in one station. No. 1 sister comes on duty at 7 A.M. and remains till 2 P.M. No. 2 comes on at 2 P.M. and stays till 8 P.M., or 9 P.M. when there is anyone very seriously ill in the ward. No. 2 again comes on the next morning at 7 A.M., while No. 3 is doing night duty from 9 P.M. to 7 A.M. Night duty we take for a week in turn. During the term of five years the sister is allowed two months' privilege leave on full pay. She can also occasionally get (if convenient) 10 days' station leave, and sometimes even three days' district leave is given. Sick leave, up to a maximum of six months, is allowed during the term of five years. This leave must be taken in India. For each ward, with an average of 25 beds, there are two orderlies. The orderly relief is changed every six hours, and in most stations there are four reliefs. Sometimes when special orderlies are required, there are as many as 18 or 20 doing duty in the wards where the sisters work. Before the orderly gets his certificate he is put through a course of stretcher drill by the medical officer, after which (if he passes his examination) his nursing certificate is given, signed by the medical officer and the sister in charge. One great difficulty in training orderlies is the little time one sometimes has to do it. Frequently from stations where no sisters are sanctioned men are sent from the regiments and are expected to be efficient nurses at the end of three months. This, clearly, is impossible, and the certificates are not worth much. Now, in stations where there are sisters the orderlies are generally allowed to remain for quite twelve months, only being called in to the regiment once for about a fortnight for musketry training. At the end of the year, if the man is intelligent, conscientious, and fond of his work, it is surprising how capable a nurse he makes. I have met a few most excellent. If he is not a suitable man in every way he can always be returned to the regiment and another man sent in his place. In addition to the practical training in the wards the senior sister holds a class about once a week on the general principles of nursing. Very often orderlies remain three, and even four, years in the wards at their own request. Native servants do the roughest of the work in the wards. I think it is quite impossible to point out the great good done by the influence of women, both technically as nurses and as ladies, and the good tone introduced by nurses fresh from the perfection of management of a civil hospital at home. From my own experience I find the orderlies much better and more willing to learn than I ever expected. I have seen them infinitely gentle when handling a sick comrade, and soldiers, when sick, behave most splendidly, always grateful and cheerful. In our wards all the cases are acute; when convalescent they go to the other wards; when chronic, they come home to England. When there are many cases and the work is heavy (which, by the way, is almost always in some stations) the sister, in addition to her ordinary duties, has just got to help the orderlies, and sponging patients, with the thermometer 112° in the shade, is no easy work, and you can imagine how persistently one has to sponge or ice-pack in a hot climate, and how imperative it is in cases of heat-stroke and fever. Yes, after some months of this work one does so long for the delights of the cool Himalayas; and with what a sigh of relief one wakes up the first morning of the 60 days' privilege leave. It is astonishing how many of the orderlies prefer remaining in the furnace below to what they describe as "climbing them khuds" (khud means a mountain side). The suggestions I should make are: (1) That a messing allowance be granted; (2) That the number of the sisters be increased so that no military station is without them; (3) That the sick leave might be extended to leave in England, or a sea voyage, if the medical officer considers it essential; the Government to provide the passage both ways. At the present time our sick leave must be taken in the country, and I think we all agree that India is not generally chosen as a health resort. At the same time our service is young, and the Government has made many reforms, and scarcely a year passes that one does not find some little alteration for the better, and I am sure in time it will be almost perfect. Our quarters are always large and comfortable, the pay is good, the amount of leave most generous. There is a pension at the end of our service, and there is that home feeling which we have in our quarters surrounded by our own little gods. We can keep a pony, trap or bicycle, and can have our live pets about us. This, to an animal lover, means a great deal. When off duty we can potter around in the garden, play tennis or any other game we like; golf is a favourite amusement, and I think a good canter across country is about the best medicine for nurses I know of; after it, too, a nurse goes on duty so fresh. Now, I am afraid you will all be a wee bit disappointed at my paper, but it is quite impossible to explain everything to you unless you all come out to India. I cannot expect you to believe, for instance, that the thermometer can drop 30 degrees in 30 seconds, though this is a fact.

### Congress Resolutions.

The following resolutions were adopted unanimously by the Congress:—

"Whereas, the nursing of the sick is a matter closely affecting all classes of the community in every land;

"Whereas, to be efficient workers, nurses should be carefully educated in the important duties which are now allotted to them;

"Whereas, at the present time there is no generally accepted term or standard of training, nor system of education or examination for nurses in any country;

"Whereas, there is no method, except in South Africa, of enabling the public to discriminate easily between trained nurses and any ignorant persons who assume that title,

"And whereas, this is a fruitful source of injury to the sick, and of discredit to the nursing profession;

"It is the opinion of this International Congress of Nurses, in general meeting assembled, that

"It is the duty of the nursing profession of every country to work for suitable legislative enactments regulating the education of nurses and protecting the interests of the public, by securing State examination and public registration with the proper penalties for enforcing the same."

A second resolution was adopted protesting against the sending out of pupil nurses to private duty during their period of training in the schools.

### Nurses' Day.

Saturday was "Nurses' Day" at the Pan-American Exposition, when the entire Congress was entertained by the managers. A mass meeting was held in the Temple of Music at one o'clock, at which Miss McIsaac presided. Mayor Conrad Diehl, who has been a practising physician, welcomed the delegates to the Exposition and congratulated them upon the success of the Congress.

Mrs. John Miller Horton, president of the committee on entertainments and ceremonies representing the board of women managers, also welcomed the Congress, saying: The request to say a few words of welcome to you came to me as a pleasant means of expressing the deep interest I feel in your organisation, and my appreciation of the noble work in which you are engaged. Some of you have come to us from over the sea. To you I extend a double welcome. It must be remembered that only a few short years ago you were obliged to fight the prejudices that new methods are apt to encounter in the Old World, and those of us who were travelling in foreign climes were obliged to telegraph to London for help in dire emergencies. It will be recalled that the uniform you wore then sent terror to the hearts of many of the gay revellers at Mediterranean resorts, who thought only of themselves and did not wish to have any reminder of the grim presence of disease. To me that uniform is a badge of honour worthy to rank with the cross of St. Louis or Victoria. Your profession calls for not only great technical skill but a rare patience and courage. No words can express the gratitude we feel towards the noble self-sacrificing women who come to us in our sorrow and anxiety for the loved ones. They are indeed ministering angels, bringing healing in their wings. "Most cordially I bid you welcome, thrice welcome. May your coming here be fraught with as many blessings to you, as has the coming of your sisters been to those of us who have had the comfort and the pleasure of having you abide with us in our homes and in our hearts."

This was suitably responded to by Miss McIsaac; and Mrs. Bedford Fenwick having addressed the meeting on "The Higher Education of Trained Nurses,"

Miss Louisa Stevenson, of Glasgow, a favourite speaker at the Congress, offered the congratulations of the National Union of Women Workers of Great Britain and Ireland, which she represented.

A paper entitled "A Retrospect and a Forecast," was read by Miss C. J. Wood, of England.

The Congress closed by singing "America," with music by Brooke's Marine Bands of Chicago.

The social side of the Congress was a feature of the great meeting. Monday afternoon Miss Damer, of Buffalo, gave a reception at her home in West Mohawk Street for the delegates; on Tuesday the members of the Spanish-American War Association entertained sixty guests at dinner at the Woodbine in Johnston Park, at which Dr. Anita Newcomb McGee did the honours; on Wednesday the officers of the Women's Union, in whose building the Congress was held, received in honour of the nurses; and on Friday evening the Buffalo nurses' societies gave a reception at Castle Inn in Niagara Square, where the foreign delegates were entertained.

### The Nurse's Never.

By a Medical Lecturer on Nursing.

(*Continued from page* 7.)

Never become a nurse unless you have some special fitness for the work. "It seems a commonly received idea among men and even among women themselves, that it requires nothing but a disappointment in love, the want of an object, a general disgust, or incapacity for other things, to turn a woman into a good nurse."

Never wear creaking boots or a rustling dress in a ward or sick room; they are unfashionable.

Never walk stealthily or weakly. Always be firm.

Never speak in a whisper in a ward or sick room.

Never disinfect your hands with mercurial or carbolic lotion, unless ordered to do so. Wash your hands in warm water with soap, and when you have made a good lather, pour about a teaspoonful of turpentine over them and wash with this. Never put the turpentine in the water before you begin to wash. This is a waste of turpentine, and you don't produce a proper lather.

Never scrape the part of the finger underneath your nails. Cut the nails squarely across with the scissors or penknife, and then use a nailbrush.

Never neglect your teeth. Use a soft brush with soap powder at least night and morning, and rinse out your mouth with a solution of one dram of Calvert's Carbolic Acid No. 2 to a pint of water.

Never ventilate a sick room from an adjoining passage. A change of air may not be fresh air.

Never confuse cold with ventilation.

Never fear to open a window widely, it is the draughty half-inch that does the harm.

Never allow any door or window to rattle or creak.

Never stuff up a chimney or fireplace. It makes an excellent ventilator.

Never hesitate to open a window at night. Night air is always the purest.

Never leave a patient in a sick room when the floor is damp or drying.

Never hang your house-thermometer near the fire or the door. Hang it at the head of the bed or in the middle of the room.

Never make a fire with all the "kindling" below and the coals on the top. All fires should be lighted from near the top.

Never use a poker to stir a fire with if you can find a stick.

Never shovel coals on the fire if your patient is disturbed by the noise. Have the lumps brought in wrapped in paper and put them on the fire with your hand.

Never allow gas to escape when lighting a gas jet.

Never turn down the wick of a kerosene lamp, but shade the light. A turned down wick wastes oil and causes a bad smell.

Never allow a candle to smoke after it is blown out.

Never place a candle on a bed or anywhere near cotton wool. A woollen Father Christmas has more than once been the occasion of a coroner's inquest.

Never strike a light, or put your hands near a flame, when they are wet with spirit.

Never allow a light to come near benzine or ether.

Never keep flowers in a ward over night. It makes the ward look much fresher to have them brought in in the morning.

Never cook anything at the patient's fire.

Never bring a slop pail into a sick room.

Never depend on fumigations to purify the air.

(*To be continued.*)

### The Care of Epileptics.

HINTS FOR PRIVATE NURSES BY A TRAINED NURSE. (*Continued from page* 13.)

The normal daily diet of the patient should be carefully considered and watched. In some cases a troublesome fancy for particular and most unsuitable foods will have to be dealt with. Many epileptics have large and abnormal appetites added to somewhat abnormal cravings for the special types of food which prove by experience to be most harmful to them. An indigestible meal is almost sure to be followed by a fit. And it is most desirable in dealing with patients whose attacks come on chiefly during the night to arrange that the principal substantial dinner is taken at mid-day. A light last meal is essential to the welfare of the "night fit" type of case. While epileptics as a class do well on a light unstimulating diet with limited quantities only of animal food, it must be borne in mind that many such individuals need a considerable amount of nourishment. An extended experience in these cases has convinced me that fits may be induced and aggravated almost as much by under-feeding as by over-feeding. Many patients of a pale neurotic type, run down very low through long living on a too light diet, become excitable and nervous and are thus rendered more sensitive to the exciting causes which lead to fits.

### The Neurotic Type.

The dull, apathetic epileptic of slow and sluggish temperament may safely be kept on a somewhat chronic *régime* of under feeding. But the neurotic, excitable, and emotional type can dispose of a more generous dietary and appear to be less liable to fits when well fed. It is hardly necessary to emphasise the fact that even when the diet is permitted to be somewhat generous, all foods given to an epileptic should be of a light and digestible nature. Nutritious food does not need to be either heavy or rich. Pastry, cheese, nuts, made dishes, and foods accompanied by rich seasonings and sauces are best avoided. General diet rules will be laid down by the doctor. The nurse will have to take care that the details of the diet fit in with the general plan. Where red meat is allowed—though it is entirely forbidden in many cases, and the patient put on a purely vegetable *régime*—it should preferably be taken at the mid-day meal. The evening dinner is best made up of soup, fish, and a light pudding, with a small quantity of ripe fruit. Chicken, or a small piece of pigeon or light game may be given occasionally instead of fish.

### Troubles of Constipation.

Constipation is often a troublesome complication in epilepsy. The loss of nerve power and general want of constitutional vitality extends to the intestines, and the bowels in consequence prove torpid and inactive. The nurse must guard carefully against constipation, and a properly arranged dietary may serve to correct the necessity which otherwise arises of giving aperients regularly. Brown bread, prune jellies, stewed Californian plums and fruits, honey, figs, and porridge should all be employed to keep up activity of the bowels. One day's constipation may give rise to a series of violent fits, so that the state of the bowels is a very important item in the nursing scheme of an epileptic. Stimulants are rarely prescribed save in a case accompanied by extreme anæmia, when an occasional glass of Burgundy is found useful. The open-air cure is as beneficial to epilepsy as it appears to be in consumption. An open window at night—so arranged as to preclude direct draught—should be insisted upon. As a rule these patients do not "take cold" readily so that fresh air may be administered in large doses. Cold bathing is not generally suited to cases of epilepsy, since few such patients appear to possess the re-active power necessary to the use of the cold bath.

### After Seizures.

The patient after a seizure should be kept lying down with very loose clothing for at least two hours. And it is advisable—more especially where the eyesight is defective or the attacks appear to follow exposure to light or sun glare—to keep the room extremely dark. If it be found that exposure to strong sunlight predisposes to attacks, it is safer in the summer months for the patient to wear smoked glasses. If glasses are not allowable owing to the risk of injury, should the patient fall whilst wearing them, a so-called "sun umbrella"—lined with green—should be used when the patient exercises in the sunshine. After a succession of fits it is usually thought necessary to confine the patient to bed for 24 hours or longer. Many epileptics have seasons of specially bad spells when for no accountable reason fit after fit of an unusually violent nature takes place. This is specially true of patients whose attacks generally take place periodically. If the periodicity becomes broken, a series of fits of a specially bad type often set in. A week's modified rest-cure in bed is usually ordered in such cases to relieve the patient of all brain and nerve worry and excitement.

### Healthy Occupations Needed.

A tendency to magnify trifles into big misfortunes, is a nervous trait common to the epileptic. A nurse should strive to counteract morbid mental tendencies, should banish unwholesome sensational literature from her patient's library and discountenance the reading in newspapers of accounts of murder trials and cases of suicide. It is half the battle in epilepsy to keep the patient's mind from morbid broodings and fancies. Encourage them to take an interest in current events, and try to so occupy their attention that they have little time for unhealthy introspection and self-pity. Some epileptics merge very closely on the dividing line between sanity and insanity, and healthy occupation may save them from crossing the border.

### Suggested Hobbies.

A love of "collections" is a capital hobby for these patients. Stamps, crests, pictorial postcards—anything that will captivate their attention. The writer has found the postcard craze a source of keen happiness. During acute periods when it may be necessary for the patient to remain in bed for some time, their postcards carry them pictorially on long travels, and fill up many otherwise dull hours. To arrange them in an album is interesting without being tiring or exciting. Scrap-book making for hospitals, a collection of autographs, getting together in the summer an herbarium of dried flowers and ferns and arranging these in winter, are all attractive and engrossing occupations. An inventive nurse may enlarge on these hobbies so as to make perpetual change and a wide horizon of interest. Epileptics are too apt to regard themselves as a class set apart, to whom the average avenues of usefulness and happiness are closed. It is a pleasant revelation to them to find that they have their niche in life, and that there are many ways in which their fingers may be made of practical use to others and of interest to themselves. For bad cases whose spells in bed are long and frequent, the game of patience proves an excellent hobby. There are many easy developments of the game, needing no mental strain, which can be played alone. With a bed-table and a pack of cards an epileptic may pass pleasantly many otherwise monotonous days in the bedroom.

### Echoes from the Outside World.

### AN OPEN LETTER TO A HOSPITAL NURSE.

Although it has been known in official circles for some time that the Ameer of Afghanistan was in failing health, his death was somewhat sudden. The Ameer, conscious that his life was a precarious one, did his best to ensure peace after his death, by arranging that all should swear fealty to his eldest son, Habibullah, now nearly 30, during his lifetime, and by giving him ample instructions as to his behaviour. But it will need a strong man to take Abdurrahman's place, for he ruled with a rod of iron, punishing and killing as he thought fit. He had a chequered career. Till he was 18 he spent his days in playing pranks and making guns, then he married, and for his wife's sake, so that he might correspond with her, he learnt to read and write for the first time. Although he had a large harem, Dr. Lilian Hamilton — who was lady doctor at the Afghan Court for some time — relates that he once remarked to her, "A man may marry many times for convenience or otherwise, but his heart only knows one wife. My one wife was my first." After the death of Dost Mahomed, Abdurrahman's grandfather, the throne was taken by Shere Ali, and prolonged civil war ultimately ensued, the late Ameer being driven forth and living for 11 years in Russia as an exile. Then his chance came. The successor to Shere Ali, Yakub Khan, allowed Sir Louis Cavagnari to be massacred and was deposed. Whereupon Abdurrahman assembled a force, marched into Afghanistan, and in time restored order to the troubled land by his sheer force of will. The Afghans are a most superstitious race, and it is said that a set of false teeth made for the Ameer contributed as much as anything to impress his subjects. He used to remove them in the presence of his lieges, clean and polish them, and solemnly put them back again to the great admiration of all around who marvelled that any king should be so great that he could take himself to pieces and put himself in order again before the very eyes of his people.

---

President Roosevelt is as yet so little known by English people that any particulars about him are especially welcome. As a youth he was exceedingly delicate, pale in face, thin in body, and with a round-shouldered stoop. He determined that for the time nothing was so important to him as to obtain strength. So he took medical advice as to the kind of exercises which would be most likely to be beneficial to him, and worked at them persistently till at last he became an athlete. Early in life he began to qualify for his political career, and in 1881 he was elected a member of Assembly. He found himself in a hotbed of legislative corruption, and at first unobtrusively, but quietly and steadily, made war against it. He was smiled at by many and sneered at by others as a sort of moral good young man whose youthful zeal would soon expend itself. But it was not long before the sneers and the smiles gave way to admiration of the honest downrightness of the man's character. Then he began to make headway, and his fight for reform was crowned with success. At this stage he sustained a crushing blow—he lost both mother and wife in one week. Mrs. Roosevelt, his second wife, and hostess at the White House, has a face as remarkable as her husband's and one so striking that it will not be easily forgotten. She has brown hair and eyes which have a happy knack of laughing; her figure is still young and girlish and she carries herself remarkably well. In these days it is worthy of remark that she rarely, if ever, wears a large hat and is generally to be seen in a small bonnet. There are six children altogether, the two youngest being boys, and Mrs. Roosevelt's eldest stepdaughter, Alice, is to be introduced into society this year.

The Church Congress at Brighton last week discussed the knotty point of how best to deal with the rough juvenile element, popularly called "Hooligans." The first speaker declared that the only hope was in State intervention, and the Rev. Wilson Carlile of the Church Army believed that the evil was largely due to the indiscriminate smoking now practised by boys of all ages. He thought that if cigarette slots were prohibited, and the supply of tobacco to boys under sixteen years of age made a penal offence, a step would be taken in the right direction. The Archbishop of Canterbury was strongly opposed to any endeavour to legislate, because, he stated, "all experience tended to show that all coercive legislation was ineffective," and went on to say that the best results had hitherto been achieved by municipalities. Archdeacon Sinclair finished the debate and his speech left a particularly pleasing remembrance behind it, for he had good to say of the street ruffians. He urged that they were nothing new, that in the past they had behaved far worse than they did now; that they were only ½ per cent. of the population, and that properly trained these lads made fine soldiers. One of the members of a Shoreditch club for street lads was wounded at Spion Kop and left for dead. A comrade, also badly wounded, was lying near, groaning terribly. The Shoreditch lad cried, "Do try to die decently." It is pleasant to be able to add that both survived, and that the lad from Shoreditch won a Victoria Cross.

---

Although the American boat won the third of the five races which were to have been sailed to decide the championship, and therefore keeps the cup, the steady improvement made by *Shamrock* after the first trial was so marked that one cannot help feeling that if yet another contest remained, the chances would have been that the English vessel might have been the conqueror. The second race had been so close that over a 15-mile course there was less than two minutes difference between the two competitors. The third race was nearer still, for the victory was won by two seconds only, and Sir Thomas Lipton will probably be found next year quite as eager to uphold England's glory as he has been hitherto. And as none but the brave deserve the fair, the fairest of good fortune may possibly reward him then.

---

It is an old saying that one half of the world has no idea how the other half lives; therefore, probably, few of you have ever heard of the Church of All Hallows, London Wall, ancient as it is, which gives shelter and warmth to some 200 women and girls every day in the year except Sundays. These girls come up from the suburbs by the workmen's trains because their salaries are so slender that they cannot afford to travel by any other means, but the result is that they arrive in town an hour and a half, and sometimes two hours, before the opening of the houses of business where they are employed. This long interval had to be spent in tramping the streets, till it occurred to the Rev. S. J. Stone, the former rector of All Hallows, to have his church warmed and lighted for their accommodation. They are allowed to bring their needlework, plenty of books are provided for them to read, and a short service takes place at half-past seven. This work has been going on for two years, and the present rector, the Rev. Montague Fowler, who is carrying on with great vigour the work started by his predecessor, is anxious to extend the same hospitality to men. During the summer this has been done by means of a tent erected in the churchyard, and the numbers have steadily grown from six rather shy guests the first day to nearly 100. But the days of open tents are passed, and Mr. Fowler is anxious to provide a more permanent shelter. The site is ready to hand; all that is needed is £1,000 to build a comfortable, well-warmed, and well-lighted room with a library attached. If everyone who has personally experienced the weariness of waiting for hours in the wind and rain would give a little, the amount required would soon be made up.

### Everybody's Opinion.

[Correspondence on all subjects is invited, but we cannot in any way be responsible for the opinions expressed by our correspondents. No communication can be entertained if the name and address of the correspondent is not given, as a guarantee of good faith but not necessarily for publication, or unless one side of the paper only is written on.]

### SUDDEN DEATH OF A PROBATIONER.

"Lydia A. Beaty," Craner Hall, Leeds, late matron of Stockton-on-Tees Hospital, writes: With reference to the death of my late probationer, Miss Eveline Radford, Miss Bemrose, with whom she was staying at the time of the very sad event, was charge nurse at Stockton-on-Tees Hospital for a short time, not matron.

### NURSES AND THE CORONATION.

"L. A. S." (Coventry) writes: If anything should be done to enable Pension Fund Nurses to see the Coronation, I shall be very happy to pay whatever sum you think right.

"A. T." (Manchester) writes: I am quite sure many nurses working in the provinces would be most grateful if any arrangements could be made which would enable us to see the Coronation Procession, and would be only too glad to fall in with any suggestions.

"M. E. A." (Keighley) writes: I have seen the paragraph in last Hospital about securing seats for Pension Fund Nurses to view the Coronation. I should be pleased to join in any plan for that object, if not too expensive.

"S. G." (London) writes: I have seen the notice in Hospital respecting "Seats for the Coronation." I think it a very good idea, and would suggest that the Royal Pension Fund should erect a stand for their members, and the Hospital a stand for the "R. B. N. A.," the Queen's Jubilee, and their own subscribers. I shall be very glad to pay a moderate sum for a good view of the procession.

"K. M. T." (Greenhithe) writes: I should be very glad to pay my share and avail myself of an opportunity arranged by you to witness the Coronation procession. I am a Queen's nurse, but as the committee I am working under are not affiliated, I should not be eligible for any seats that might be procured for the Institute.

"L. F." (Dover) writes: I write to tell you that as a member of the Royal Pension Fund for Nurses I should be very pleased to pay a moderate sum, if by that means I could obtain a glimpse of the Coronation Procession.

"H. F." and "A. E. B." (Maidstone) write: Having seen that a movement is on foot by which arrangements may possibly be made to secure a sight of the Coronation Procession of our King and Queen by nurses, we beg to say that we should be delighted if the plan could be carried into effect. We could not afford much, but would be glad to know what would be considered a moderate sum, and if we can meet it we would gladly pay in advance. With many thanks for the kind interest that is shown on our behalf in your valuable paper to nurses of the R.N.P.F.N.

"F. M. W." (Harrow-on-the-Hill) writes: With regard to the remarks on "Nurses and the Coronation" in The Hospital of this week, I am writing on behalf of my colleague and myself—both Pension Fund district nurses—to say that we should be only too glad to avail ourselves of any arrangement it is in your power to make so that we might obtain a sight of the Coronation Procession. If a large number responded, could the privilege be obtained for about 10s. each? Trusting that your kind efforts on our behalf may meet with success.

"M. C." (Windsor) writes: Having seen in last week's Hospital that you will be willing to help the R.N.P.F. Nurses to see the Coronation, I shall be glad to avail myself of the opportunity of doing so. It is very kind of you to take the trouble on our behalf, and I am sure that if you can possibly arrange it for us, without leading us into bankruptcy, we shall all be very grateful to you. I know how difficult it must be, and but for your kind offer I and many others of my profession would not have thought of trying.

"A. M. H." (West Kensington) writes: Should you kindly make any arrangements for the members of the "Royal National Pension Fund" to view the Coronation procession I am quite willing to advance a small sum (if required) to procure a seat.

### NURSES' READING SOCIETY.

"Miss Moberly" writes: I am sorry to say that there has been a great falling off of members since the Society began two years ago. I had had serious thought of bringing it to an end now, but I have decided to keep it on for another year, to see whether any fresh accession of members will help to give it a new lease of life. In consequence of the reduction of funds, and also because a lady who subscribed last year has not yet done so this, the prizes will be smaller than they were last year, and only four can be given instead of five. If, however, the subscription comes in during the autumn, the prize-winners will receive additional money. The following are the names of the winners, with the number of their marks:—Miss J. Till, 395 marks, prize 10s.; Miss Lockwood, 375 marks, prize 5s.; Miss Marsh, 360 marks, prize 5s.; Miss H. Dendy, 355 marks, prize 5s. Statement of accounts, 1900-1901. Subscriptions received from members, £5 15s. Secretarial expenses, stamps, etc., £4 12s. 6d.; prizes, £1 2s. 6d. Total, £5 15s.

### THE SCARCITY OF NURSES IN WORKHOUSE INFIRMARIES.

"P. S." writes: I have been very interested in the remarks made in your columns *re* "Scarcity of Nurses at the Workhouse Infirmaries." On reading the letter of "A Poor-law Nurse," I can only think that she is either speaking of poor-law nursing as it was many years ago, or that she has had but a very limited experience. I worked for nearly four years in a workhouse infirmary, and during that time not one nurse did I meet to whom could be applied one of the adjectives that "A Poor-law Nurse" makes use of with reference to infirmary nurses. On the contrary, all I met were "bright, intelligent women" with a thorough love for their work. I have since been nursing in a general hospital, and can only say that, after having received my own training in a workhouse infirmary, I would advise anyone wishing to obtain a good "practical and theoretical" training and a "certificate worth having" to gain it by "conscious merit" in a workhouse infirmary training school.

"E. A.," ward sister at Lambeth Infirmary, writes: I have read with great interest a short account by "A Poor-law Nurse" of workhouse infirmary nursing in last week's issue of The Hospital. She must indeed have had many years' experience in such work; in fact, an experience which carries her back into the days when "Sairey Gamp," slow, ignorant, and underbred, after taking "a drop from the bottle on the chimbley" when she felt "dispoged," settled herself comfortably with the patients' pillows and sank into the arms of Morpheus. Your correspondent asks, "What inducement has a good nurse to go amongst such women?" Surely one would answer "None." And, moreover, one would add, do not associate with them, do not make them your friends. Why should one go where the training is poor, and one is only taught to nurse cases which are of everyday occurrence, and where the orders of mere sisters must be obeyed? Why go where it depends upon your own exertions as to whether you succeed or fail? Is there any need to go to a country infirmary with no training school attached? Finally, I might add, look to yourself and see before you join that you are by birth a gentlewoman, and inquire of your matron whether she is respectable, sober, honest, bright, and intelligent. (She will gladly answer all inquiries.) And if you cannot find this standard of requirements in any poor-law nursing institution the remedy is simple, enter a hospital where the real "nursing" is done by the students and the sisters carry the ink-pot."

"Another Poor Law Nurse" writes: I think it a pity that a nurse who apparently knows so little of the larger infirmaries should be allowed to write condemning them in the way I read in your columns last week. However, it makes one want to repeat what is doubtless well known in the nursing world, for it has been advocated in nursing papers for so many years past, *i.e.*, the world must recognise that, on the grounds of common humanity, the sickness and infirmity of the very poor must demand for them the same privileges and the same care as the sickness and infirmity of their more fortunate brothers and sisters, therefore, an infirmary is not of necessity a workhouse, and as an infirmary is not a workhouse, it must have a separate administrative staff of its own. Good and zealous probationers will not consent to be trained by a woman who has had no training herself. There is useful work to be done in workhouses, of course, and no "work that is pure in its purpose and strong in its strife" can be wasted anywhere, but it is not training in sick nursing. That must be in infirmaries and hospitals alone, and must be entirely disconnected with the workhouse. Happily, in some of the larger unions this is already accomplished, and may the day be not very far distant when even the quite small ones will have their own little infirmary for the care of the sick and infirm of the parish. There will be no "scarcity" of nurses then, the needs of the very poorest of our brethren will find a ready response, and the tramp and the vagrant, however low he may have fallen in the social scale, will find that he has a sister ready to tend him as gently as she would the richest of the land. From my own experience in one of the best ordered infirmaries of this country, which is entirely separate from the workhouse proper, I can state that there is certainly no scarcity of probationers there, as I believe every vacancy is filled up months before it actually occurs, and the lives of the nurses in this institution are happy, zealous, and most devoted.

### Appointments.

Brierley Hill Infectious Hospital.—Miss J. Childs has been appointed nurse. She was trained at the General Hospital, Birmingham, and has since for 10 years been nurse-matron at the City Hospital, Birmingham.

Bromley Cottage Hospital, Kent.—Miss A. Clark has been appointed as senior staff nurse. She was trained, and has since been ward sister for two years, at the Bolton Infirmary.

Brownlow Hill Workhouse Hospital, Liverpool.—Miss Margaret S. Whitson has been appointed night superintendent. She was trained at Liverpool Workhouse Hospital, and has since been night superintendent at St. Mary's Hospital, London.

Burghmuir Hospital, Perth.—Miss Louise M. F. Quilter has been appointed staff nurse. She was trained in the New York City Training School for Nurses.

Chester District Nursing Home.—Miss Bateson has been appointed lady superintendent. She was trained at the Infirmary, Birmingham, and has since been staff nurse at the Sheffield Nursing Institution, and lady superintendent of the District Home at Wolverhampton.

Cork Street Fever Hospital, Dublin.—Miss Marian Wilkinson has been appointed assistant lady superintendent. She was trained at Leeds General Infirmary.

Easington Workhouse Hospital.—Miss Caroline Anne Jackson has been appointed superintendent nurse. She was trained at Bradford Union Infirmary for three years, and has since been nurse in the same institution, at the City Hospital, Bradford; the Bradford Eye, Ear, and Throat Hospital; and the City Hospital, Birmingham.

Forfar Infirmary.—Miss Margaret A. Smith has been appointed matron. She was trained at the Aberdeen Royal Infirmary, and has since been staff nurse in Chalmers' Hospital, Banff.

Royal Boscombe Hospital, Bournemouth.—Miss Nora Langley has been appointed staff nurse. She was trained at the Royal Infirmary, Halifax.

St. Alban's Union Infirmary.—Miss Jessie Isabella Smith has been appointed head nurse. She was trained at Aberdeen Royal Infirmary, and has since been sister at Poplar and Stepney Sick Asylum; charge nurse at Park Fever Hospital; and Sister at the Royal Military Hospital, Athens.

Southwark Infirmary, East Dulwich Grove, S.E.—Miss E. G. Massey has been appointed first assistant matron. She was trained at the North Staffordshire Infirmary, Stoke-on-Trent; was sister at the Royal Infirmary, Derby; sister and subsequently second-assistant matron at Southwark Infirmary.

### Presentations.

Bournemouth Sanitary Hospital.—Miss A. Gascoigne, matron of the Bournemouth Sanitary Hospital, has been presented with a testimonial. The chair was taken on the interesting occasion by Dr. P. W. G. Nunn medical officer of health, who in making the presentation in the name of the hospital, alluded to Miss Gascoigne's approaching marriage with Mr. A. Godwin Pratt, and then paid a warm tribute to the services rendered by her during the last nine years. The gifts consisted of a gold bracelet set with turquoise, and a gold heart, from the nurses; earrings and a scarf-pin from the servants; and a basket with ferns from the porter and his wife. The presentation took place in the dining-room of the nurses' house, amid many manifestations of enthusiasm, and Mr. Pratt's answer to Dr. Nunn's address was extremely well received.

### Death in Our Ranks.

Smallburgh Workhouse.—We regret to learn of the death of Miss Florence Louisa Miller, a nurse at the Smallburgh Workhouse Infirmary, from peritonitis. At an inquest on the body last week, Dr. Williams stated that he thought the disease had been accelerated by the habit of deceased in lacing her corsets too tightly. Miss Miller was only 26.

### To Nurses.

**We invite contributions from any of our readers, and shall be glad to pay for "Notes on News from the Nursing World," or for articles describing nursing experiences, or dealing with any nursing question from an original point of view. The minimum payment for contributions is 5s., but we welcome interesting contributions of a column, or a page, in length. It may be added that notices of entertainments, presentations, and deaths are not paid for, but, of course, we are always glad to receive them. All rejected manuscripts are returned in due course, and all payments for manuscripts used are made as early as possible after the beginning of each quarter.**

### For Reading to the Sick.

TIME AND ETERNITY.

The sun had set in glory; clouds of gold
Were fringed with wondrous purple; crimson bars
Reddened the foaming billows as they rolled,
Till from heaven's blue gleamed out the silent stars.

. . . . . . .

And while I gazed I wondered what might be
The new, diviner Land for which we wait;
For earth itself, from stain of evil free,
Would gleam with glory from the Golden Gate.

. . . . . . . . .

But there no clouds shall gather, and no more
The ocean rage—emblem of deep unrest;
No storms shall sweep across that radiant shore,
No night shall shroud that City of the blest!

This earth is beautiful; o'er land and sea
The mighty shadow of God's thought is cast,
But brighter far the Home that is to be,—
O Christ! receive us to that Home at last!

*R. H. Baynes.*

We must do common things with a simple heart and high intentions. We must step boldly through the night with our eyes on the morning. This is the life of faith; this is the foundation of a Christian character; this is following Jesus; this is guiding the path of our progress by "the Light of Life."

It is just when we link high thoughts with common duties that we are most truly Christians. It is just when the soul learns some devotion to Him Who is the Meeting-point of time and eternity, the Alpha and Omega, the Beginning and the End, our God and Brother—just when it strains the eye of longing and contemplative hope to the things of eternity, and then turns its hand to the rude and common duties of daily life, and the exercise of practical virtue, of purity, kindness, justice, considerateness, truth—that our work is done best, and our spiritual life grows most truly, and our probation is best fulfilled, and men are helped and God is glorified, and we rise to the true dignity of our being and are more like true followers of the Crucified, and "walk in the Light of Life."

Let us live for eternity. Let us look more steadily and constantly through what appears, to what shall always be. Blessed are ye, immeasurably blessed, if ye have grace in a world of shadows to fix your eyes on the vision of eternity.

*Knox Little.*

So when we near the shore at last,
Life's partings and life's billows past,
And in the soft'ning distance see
The haven where our souls would be,
The heart that God hath touch'd forgets
To waste itself in vain regrets
O'er griefs endured—for joys foregone—
Its day of brief existence done;
Remembering that in the light
Of God's Great Day man's day is night;
And night far spent, God's day at hand
Awaits us in the Sun-less Land!

*J. S. B. Monsell.*

### Notes and Queries.

**The Editor is always willing to answer in this column, without any fee, all reasonable questions, as soon as possible.**
**But the following rules must be carefully observed:—**
**1. Every communication must be accompanied by the name and address of the writer.**
**2. The question must always bear upon nursing, directly or indirectly.**
**If an answer is required by letter a fee of half-a-crown must be enclosed with the note containing the inquiry.**

*Recommendation.*

(15) We have to thank R. M. S. for recommending a boarding house in Glasgow.

*Epileptic.*

(16) Can you let me know how it is possible to place a girl who has epileptic fits in the Epileptic Colony?—*Nurse L. L.*

Apply to the secretary, The National Society for the Employment of Epileptics, 12 Buckingham Street, Strand, W.C. The colony is at Chalfont St. Peters. The girl seems eligible for the Meath Home of Comfort for Epileptics, Westbrook, Godalming.

*Consumption Nursing Abroad.*

(17) Will you kindly let me know the names of a few sanatoria abroad where two nurses with a year's training in consumption nursing could obtain employment?—*The "Con."*

It is impossible to give definite information on this point. Your best plan would be to write to English medical men resident in the place you wish to go to and ask for work.

*District Nursing.*

(18) Will you kindly tell me if a nurse with a three years' certificate in medical, surgical, and midwifery nursing would be able to undertake a country district without previous training in that work? Would it be advisable to join an association, or to begin on my own account?—"*B.*"

You cannot begin "district" nursing on your own account. Possibly you mean "private nursing in a country district." You are qualified, but it would be advisable to join an association until you gain experience.

*Child's Nurse.*

(19) Is there not a place called the Norland's Institute where a young lady could receive preparatory instruction before taking a situation as child's nurse?—*J. S.*

Apply for prospectus to the secretary, 10 Pembridge Square, London, W.

*Dispensing in America.*

(20) Can you tell me how to obtain a post as dispenser, or nurse-dispenser in America? I am 20 years of age, and have no money. —*Chicago.*

You will find a list of the more important American hospitals and nursing schools in "The Nursing Profession: How and Where to Train." Write to the Secretaries, state your qualifications, and ask for particulars; but you will find the post difficult to obtain, as preference is naturally given to Americans on the spot.

*Macintoshes.*

(21) Is there any hospital which has macintoshes on every bed, covering them fully?—*Nurse J.*

No. Macintoshes are only used when required. There are many spring beds, however, the covers of which are made of macintosh.

*Cookery.*

(22) Would you kindly tell me where, in or near London, I could obtain practical lessons in cookery at a reasonable cost?—*K. L. S.*

Apply to the National Training School of Cookery, 72 to 78 Buckingham Palace Road, London, S.W.

*Address.*

(23) I should be glad to know the address of the United British Women's Emigration Association.—*An Enquirer.*

The Imperial Institute, S.W.

**Standard Books of Reference.**

"The Nursing Profession: How and Where to Train." 2s. net; post free 2s. 4d.
"Burdett's Official Nursing Directory." 3s. net; post free, 3s. 4d.
"Burdett's Hospitals and Charities." 5s.
"The Nurses' Dictionary of Medical Terms." 2s.
"Burdett's Series of Nursing Text-Books." 1s. each.
"A Handbook for Nurses." (Illustrated). 5s.
"Nursing: Its Theory and Practice." New Edition. 3s. 6d.
"Helps in Sickness and to Health." Fifteenth Thousand. 5s.
"The Physiological Feeding of Infants." 1s.
"The Physiological Nursery Chart." 1s.; post free, 1s. 3d.
"Hospital Expenditure: The Commissariat." 2s. 6d.

All these are published by the Scientific Press, Ltd., and may be obtained through any bookseller or direct from the publishers, 28 and 29 Southampton Street, London, W.C.

### Travel Notes.

By Our Travelling Correspondent.

### LXXXIII.—THE FATAL FIELD OF SEDGEMOOR.

It was with the greatest delight that I accepted an invitation from my hostess to spend a long day in viewing the field and neighbourhood of Sedgemoor, and we started early with the able co-operation of "Tommy," a dun-coloured cob of character, who made nothing of the 30 miles or so that he was invited to traverse and drag with him two antiquarian enthusiasts of no fairy-like weight or proportions. Our first point was Chedzoy Church (every village there begins or ends with zoy) where the fine old buttresses show on their grey and time-worn stones the deep scars caused by the sharpening of the swords and scythes used by Monmouth's army, dispersed and ruined on the disastrous day of Sedgemoor, 1685.

A large proportion of the moorland has disappeared under cultivation, but the "Rhines," so fatal to the insurgents, still remain in their original positions, and in no wise altered in character. These "Rhines "are deep cuttings, of varying depth and width, which intersect the moor in all directions, constructed, as I understood, chiefly to drain the peat land. The largest of these "Rhines," called "The Old Bussex Rhine," engulphed friend and foe on that sad July day more than 200 years ago.

A heavy heat fog wrapped the moor in an impenetrable veil which entirely concealed the movements of the Royal troops, who, well disciplined and well led, had entrenched themselves in an advantageous position.

Monmouth's army, clustered round his blue flag, was encamped in the Castle field, which, together with a considerable part of the fighting ground, was visible from the church tower of Bridgewater, whither the panic-stricken inhabitants mounted, to follow with their own eyes the success or failure of the Protestant insurgents. Alas for their hopes! it was all failure — Monmouth had neither genius, tact, nor even courage. He moved amongst his followers with a face of gloom that was enough to depress regular troops, and which, in such an army as his own, was calculated to insure defeat. Macaulay gives a splendid and most picturesque account of this disastrous rising, with its dreadful sequel, of the tyranny of Kirke's Lambs and the iniquitous proceedings of the Bloody Assizes under Judge Jefferies.

### Weston Zoyland and Chedzoy.

I could not ascertain with certainty at which house Lord Feversham, the commander of the Royal troops, slept on the eve of the battle, but it certainly was in Western Zoyland somewhere; and it was in this church, with its magnificent tower, that 500 prisoners from Monmouth's shattered army were guarded after that disastrous day. There they were herded together with cruel crowding, a large proportion of them desperately wounded, and many of them breathed their last within the sacred walls.

In the small village of Chedzoy, in a cottage garden, stands a singular cross which I was told was in some way connected with the battle. The clergyman, however, was absent, and village erudition went no further in elucidating its history.

The churchyard and church are a marvel of neatness and good order, and the building well worth visiting, the carving being extremely fine. It is on the south side of this church that the rebels sharpened their swords and scythes. We hear of John Churchill, afterwards the Duke of Marlborough, at this battle, where his wonderful genius as a soldier began to show itself.

It is grievous to remember that the pusillanimous Monmouth deserted his ill-trained but faithful followers and positively ran away to secure his useless life—a matter, indeed, in which he failed, and it must ever remain the deepest blot upon James II.'s name the cruel malignity with which he hunted his nephew to his death, refusing all humble requests from the unfortunate Duchess to spare his life, even if he was sent into perpetual exile. Monmouth was a contemptible character one must own; but then what a bringing up he had had, and what vicious and unworthy surroundings had been his.

### Bridgewater.

In wandering through the dull and flatly uninteresting streets of Bridgewater, I found myself peopling them with the Monmouth troops and listening to their ill-beaten drums and incorrectly blown bugles, if indeed they used these last-named instruments. What a motley crew they must have been! According to Macaulay the horses pressed into the service were of all sorts, ages, and conditions, some fresh from the plough, some post-horses, some even unbroken colts, hardly one that had ever known the touch of a bridle hand or the pressure and weight of a human being on a saddle, and a large proportion so entirely untrained that they "became ungovernable as soon as they heard a gun fired or a drum beaten!"

We ascended the tower of the grand old church that we might the better realise the feelings of the wives, daughters, and sweethearts who climbed there that foggy July day to watch for the dear ones who would never again return.

Bridgewater Church, with all its tragic associations, is well worth a visit. The pew for the mayor and corporation is truly splendid. These worthies are sheltered from association with the vulgar herd in the body of the church by a magnificent oaken screen, every pillar richly carved and embossed. Over the altar is a large picture of the weeping women at the foot of the cross. The citizens ascribe immense value to this, and no doubt it is a valuable painting; but I was unable to give it adequate attention by the wrath that consumed me at the conduct of a vulgar and sacrilegious tripper who coolly deposited his hat and umbrella on the altar! Over the two porches there are small chambers open to the church which, I should think, must at one time have accommodated the choir and orchestra, or they might have been used by the squires of the neighbourhood. Outside there are some very curious tombstones with figures on them: they lie immediately under the walls of the church, and are so completely defaced and rounded by time and weather as to leave no sign by which to trace their identity.

How infinitely more fortunate were those who bravely met their death on that grim moor to that of the unfortunate leader who escaped from it only to fall into the tender mercies of his hard, narrow, and bigoted uncle, or than those equally unfortunate fugitives and their innocent families who found tyranny, torture, and eventually death at the Bloody Assizes of Judge Jefferies.

### TRAVEL NOTES AND QUERIES.

Rules in Regard to Correspondence for this Section.—All questioners must use a pseudonym for publication, but the communication must also bear the writer's own name and address as well, which will be regarded as confidential. All such communications to be addressed "Travel Editor, 'Nursing Mirror,' 28 Southampton Street, Strand." No charge will be made for inserting and answering questions in the inquiry column, and all will be answered in rotation as space permits. If an answer by letter is required, a stamped and addressed envelope must be enclosed, together with 2s. 6d., which fee will be devoted to the objects of "The Hospital" Convalescent Fund. Any inquiries reaching the office after Monday cannot be answered in "The Mirror" of the current week.

Pensions at Bordighera (Beta).—I know of nothing at such low terms as you offer. I do not think it possible to feed and lodge anyone honestly at 5 francs in the season on the Riviera. The Hôtel Beau Rivage is the cheapest place I know of, terms from 6 francs. Write, and try to make an arrangement. There are several reasonable Pensions. You might write to the Pension Canzi and the Pension Jolie. At any rate, go to one of these first, and if it is beyond your means, take a room, have your breakfast with your landlady, and your dinners at a cheap restaurant. If, however, anyone will take you for 6 francs per day, you will not improve upon such terms, I feel sure. The cheapest route is thus:—Newhaven, Dieppe, Paris, Lyons, and Marseilles, second-class return, £7 13s. 9d.; second, single, £4 4s. 9d.

